# Book Review

**Published:** 1938

**Authors:** 


### Reviews of Books

**Surgery of the Sympathetic Nervous System.** Second Edition. By G. E. Gask, F.R.C.S., and J. P. Ross, M.S., F.R.C.S. Pp. xii., 191. Illustrated. London: Baillière, Tindall & Cox. 1937. Price 16s.—So many ailments may be relieved by the comparatively new and unfamiliar operations on the sympathetic nervous system that it seems worth while to review this excellent book somewhat more fully than usual. A centre has been discovered in the hypothalamic nuclei, stimulation of which in animals causes signs of sympathetic vasoconstriction, raised blood pressure and output of adrenalin. A tumour in a human case has been described which corroborates this evidence. Clinical evidence shows that periarterial sympathectomy in certain cases does good, in spite of the theoretical difficulty in explaining why; the peripheral vasodilatation persists for three to four weeks, which may be sufficient to lead to the healing of chronic ulcers. Pain arising from vascular conditions such as causalgia, Raynaud's disease, gangrene or intermittent claudication may be permanently relieved by a periarterial sympathectomy. It is especially indicated in cases of senile or diabetic gangrene of one or two toes, without fever or toxæmia. The toes may be left to drop off, or the last strands of tissue may be snipped through. The complete removal of all nerve-fibres is facilitated by a trickle of normal saline, and the fibrous sheath in which the artery and the vein run should be excised as well as the adventitia. The artery will contract down to a very small cord when the operation has been properly performed. Although animal experiment would seem to indicate that preganglionic section of the sympathetic trunk will give more permanent results than ganglionectomy for Raynaud's disease, the authors doubt if this is so in practice. Good descriptions are given of the anterior and posterior methods of approach for removal of the stellate and neighbouring ganglia; they find the anterior route easier. Cases of Raynaud's disease uncomplicated by ulceration or sclerodermia generally give a very happy result; those with sclerodermia are seldom benefited. Lumbar ganglionectomy gives a better result than stellectomy. Thrombo-angiitis obliterans is sometimes benefited, sometimes not; it is valuable to take an arteriogram by means of X-rays and thorotrast to discover if main arteries are blocked. Polyarticular rheumatoid arthritis involving the wrists and hands or feet and ankles is suitable for sympathectomy, and at first the results may be brilliant, but there is often some relapse later. Patients with erythrocyanosis frigida of mild degree show much improvement, but those with a good deal of thickening of the ankles are less promising. To test whether sympathectomy will be good in a case of Hirschsprungs' disease, valuable evidence may be obtained by giving a spinal anæsthetic and watching the effect on a barium enema. If peristalsis of the colon occurs, there was sympathetic inhibition, and operation is likely to give a satisfactory result. Many operations have been proposed and performed: ramisectomy, which is difficult on account of the small size of the nerve fibres; left lumbar ganglionectomy; division of the presacral nerve over the sacral promontory, and the stripping of its middle and lateral roots off the left common iliac vein and the bifurcation of the aorta up as far as the origin of the inferior mesenteric artery, and baring of the proximal inch of the artery. If one goes further one is likely to damage the parasympathetic (motor) fibres derived from the sacral plexus. The last operation appears to be the best, but in male patients it may lead to sterility. Sympathectomy for cardiospasm has been disappointing. As is well known, resection of the presacral nerve usually stops the pain of spasmodic dysmenorrhæa; for vesical pain the results are disappointing in malignant cases, encouraging for long-standing painful cystitis. There is a type of renal pain associated with slight dilatation of the pelvis and clubbing of the calices which is relieved by stripping the nerves off the renal vessels. Suitable cases are those in which the pain can be reproduced by distending the renal pelvis, but it is abolished by an injection of eserine. Good results have been obtained in patients with angina pectoris, in about two-thirds of the cases, by removing the inferior cervical, first and second thoracic ganglia on the left side. The difficult ailment called "causalgia," in which after a nerve injury or amputation the patient complains bitterly of pain, and atrophic changes associated with skin red when warm and blue when cold, can usually be relieved by sympathectomy, though why it succeeds is far from clear. Periarterial sympathectomy is often sufficient, but ganglionectomy is more reliable.—A. R. SHORT.

**The Baths of Bath.** By P. R. JAMES, M.A. Pp. 176. Illustrated. Bristol: J. W. Arrowsmith Ltd. 1938. Price 5s. This is an interesting and amusing account of the early development of Bath as a therapeutic centre after the Corporation obtained possession of the monastic baths. Lavish quotation from contemporary documents and reproduction of early illustrations add much to its value.

**Studies on the Physiology of the Middle Ear.** By J. G. BYRNE, M.D., LL.D. Pp. xi, 298. Illustrated. London: H. K. Lewis & Co. Ltd. 1938. Price 18s.—The author of this book describes a series of experiments on animals in which the actual movement of the tympanic membrane in response to excitation of the tensor tympani and stapedius muscles is directly measured. There is a useful summary at the end of each chapter, and the book is well produced, illustrated and indexed.

**Surface and Radiological Anatomy.** Edited by A. B. APPLETON, M.A., M.D., W. J. HAMILTON, M.B., B.Ch., D.Sc., F.R.S.E., and I. C. C. TCHAPEROFF, M.A., M.D., B.Ch., D.M.R.E. Pp. xi., 311. Illustrated. Cambridge: W. Heffer & Sons Ltd. 1938.—The authors have produced a book which aims, by the extensive use of illustrations, to co-ordinate radiological anatomy with features which can be ascertained by external examination and by dissection. For this reason the book will be best appreciated by those who have a knowledge of normal radiological appearances. The large number of plates and figures should be a great help to the student in gaining this knowledge. The authors suggest that senior students should make greater use of other physical methods of examination. Explanatory diagrams are included for the study of the distribution of nerves by means of the Smart-Bristow coil. Short notes have been included dealing with certain diagnostic techniques such as gastroscopy, kymography and thorotrast examinations. For some reason no mention has been made of tomography. Anatomical regions are each given a separate chapter. Emphasis is laid on the normal, and great care has been exercised in the selection of suitable illustrations. These are good, although in the method of reproduction used there is a certain loss of detail in the radiographs. Radiographs are reproduced as negatives, in which form they are generally examined, and there has been no retouching. In all there are over 338 illustrations in a book of 311 pages. The index is sound and there are some useful tables at the end of the book.

**Report on the British Health Services.** Pp. viii., 430. London: P.E.P. (Political and Economic Planning). 1937. Price 7s. 6d.—The most recent volume issued by P.E.P. on the British Health Services is one of the most comprehensive reports on this subject available in this country, and will appeal to both medical and lay men alike. It is issued at a time when the campaign for using the health services is in full swing and will serve as a most useful book of reference for indicating the varied number of ways of promoting health and fitness. The report covers national, municipal and voluntary services, the term "health services" being used in its widest sense, e.g. to cover protection of food supply, control of the trade in medicine, infectious disease, industrial health, maternity and child welfare, school medical service, organization of the medical profession and medical services generally, nutrition, physical education and psychological medicine. The report is a most valuable review of the general position because it is not the work of a single individual but represents the considered views of persons drawn from many professions both medical and lay. As the report indicates, until recently medicine was largely confined to work of a curative nature and health was thought of as being merely "free from illness" but now it is something positive and implies the facility to enjoy life to its fullest extent from all aspects. In the section dealing with National Health Insurance it is natural that this should centre round the general practitioner, and advocate the general practitioner as being the pivot of the extended scheme. Some of the writers question this desirability and indicate their preference for state medical service. The report deals with proposals for future development generally, including the need for pushing forward the improvement in nutrition, particularly by extending milk schemes, and puts forward the theory that existing health services should be better used both by the public and by other health services, that National Health Insurance should be extended to cover dependants of existing insured persons and that much more research should be done on medical, social and economic causes of ill-health.

**Collected Papers on Tuberculosis.** By SIR R. W. PHILIP, M.D., LL.D., F.R.C.S., F.R.S.E. Pp. x., 460. London: Oxford University Press. 1937. Price 21s.—This volume derives its significance from the fact that Sir Robert Philip has played a dominating part in the inception and determination of anti-tuberculosis measures for half a century. He tells how his interest in the problems of tuberculosis was

"awakened beyond recall" in 1882, while on a study tour in Vienna. No time could have been more opportune for the impact of a young and vigorous mind on these problems, for the older generation of medical men was ill prepared for the new orientation imposed by the discovery of Koch's bacillus. Full of enthusiasm, Philip returned to Edinburgh, to face both active and passive opposition to any new approach to a subject regarded as already threadbare. It was in the teeth of this that he established, in 1887, the "Dispensary System," since adopted all over the world. The Victoria Hospital for consumptives followed, and as early as 1898 he put in a plea for a "tuberculous colony where poor patients might be enabled, for long periods and under hygienic conditions, to maintain themselves honourably." Notification, contact examination, isolation of advanced cases, after-care and open-air schools were all urged by him at a time when such things were not only unthought of, but aroused strong opposition when suggested. Philip indeed suffered the customary fate of the medical pioneer in his statesmanlike efforts to formulate a scheme of organized and co-ordinated operations against tuberculosis. But he had his reward ; to few is it given to see his early labours bear such abundant fruit. These Collected Papers, the first published in 1898 and the last in 1934, record his consistent advocacy of the policy and method which he first introduced in Edinburgh on a voluntary basis, and which in 1911 was adopted for the whole country when legislation was for the first time specificially directed against tuberculosis. As such they are full of interest to all who are working to improve the health of the nation.

**The Physiology of the Kidney.** By H. W. SMITH, A.B.; Sc.D., M.S. Pp. 10, 310. Illustrated. London: Oxford University Press (Humphrey Milford). 1937. Price 15s.—This review of the more recent work regarding the function of the kidney should prove most useful to those who wish to bring up to date their knowledge of this difficult subject. It is divided into three parts : the first includes the theories of renal function, glomerular filtration, tubule activity, and a very useful section on renal clearances ; the second deals with the kidney as a regulator of the composition of the plasma ; and the third contains chapters on water excretion, diuretics, renal nerves and blood flow. The discussions and explanations are clear and a considerable amount of literature has been digested ; the references cover about 500 papers since 1920. The style is easy and there is a satisfactory index. A few obvious misprints will no doubt be corrected in a subsequent edition.

**Practical Procedures.** Edited by Sir HUMPHRY D. ROLLESTON, Bart., G.C.V.O., K.C.B., M.D., F.R.C.P., and A. A. MONCREIFF, M.D. Pp. 293. Illustrated. London: Eyre & Spottiswoode Ltd. 1938. Price 10s. 6d.—This is announced as "a really practical guide to clinical procedures" to fill the gaps which modern hospital teaching has left in the knowledge of the general practitioner. Several of the chapters admirably accomplish this end. But it is difficult to believe that by the time he has qualified the practitioner has not had ample experience of blood counts, the use of the sphygmomanometer, passage of catheters and clinical examination of the urine, to give four examples: or on the other hand that the description given here of artificial pneumothorax, the spinal plaster jacket, blood transfusion, auto-transfusion for ruptured spleen, or local anæsthesia for bronchoscopy will really bring these methods within the scope of general practice. Much of the book is devoted to syringe-and-needle work—there is even a chapter on three-way syringes. And there is much repetition: for example, local anæsthesia of the urethra is described three times, while both three-way and two-way syringes are illustrated twice over. The book is pleasant to handle and easy to read, and meticulously indexed.

**Essentials of Modern Medical Treatment.** By VINCENT NORMAN, M.D., M.R.C.P., F.R.C.S. Pp. xvi., 200. Hutchinson Scientific and Technical Publications. 1936. Price 10s. 6d.—This book may be described as a précis of medical treatment. As far as it goes it sets out clearly and accurately the standard forms of treatment in various diseases, but the student who requires a more detailed description of treatment will necessarily have to consult larger works on the subject.

**Food and Physical Fitness.** By E. W. H. CRUICKSHANK, M.D., D.Sc., M.R.C.P., F.R.S.E. Pp. xi., 148. Edinburgh: E. & S. Livingstone. 1938. Price 5s.—During the session 1936-37 Dr. Cruickshank, Regius Professor of Physiology in the University of Aberdeen, gave a series of public lectures under the James Farquhar Thomson bequest. Those lectures form the basis of the book now reviewed, which deals in the main with the part played by food in the maintenance of physical fitness. Whilst recognizing that nutrition depends upon many factors besides the mere supply of sufficient and suitable food, Professor Cruickshank maintains that food is the foundation of health. Thus his book becomes another of the numerous simple expositions of foods, their composition and their utilization, with which the medical profession and the public are familiar. In some respects the work is not reliably up to date ; for example, the grading of milk as quoted is that applicable to Scotland, which differs slightly from the " special designations " laid down for England and Wales. The use of the term " physical fitness " in the title might lead to an expectation that the problems of exercise, athletics and " keeping fit " would come under consideration. This aspect of nutrition is not, however, dealt with, which is a pity, seeing how much our knowledge has recently been enlarged by the studies of the Glasgow physiologists and the writings of Dill in America. Taken as an unpretentious account addressed to the non-medical public it may convey a good deal of accurate and useful information.

**Speech Training for Cases of Cleft Palate.** By M. C. OLDFIELD, M.Ch., F.R.C.S. Pp. ii., 18. Illustrated. London : H. K. Lewis & Co. Ltd. 1938. Price 4s. 6d.—The value of this slight but attractively produced book lies in the fact that the information is given clearly and simply. It should prove an easy introduction to the possibility of help for cleft palate speech. The speech therapist and the cleft palate patient will both agree, I think, that it is not possible for adult patients to " forget their old bad habits of speech before learning the correct method of pronunciation " (page 1). If this were only possible how much easier the task of re-education would be ! The first essential is surely to learn the new method of pronunciation and then by " daily conversation practice "—to quote Miss Kingdon Ward—" gradually build up the newly-acquired method of speech." The photographs of the groups of children, showing the interesting methods used for the exercises, constitute the most valuable part of this somewhat scanty work. These should prove helpful, instructive and illuminating to both parent and speech therapist.

**Occupational Therapy : an Addendum to the Handbook for Mental Nurses.** By Miss RUTH DARWIN. Pp. 16. London : Baillière, Tindall & Cox. 1938. Price 6d.—This admirable booklet has been published in advance of the rest of the new edition of the *Handbook for Mental Nurses*, and gives in a clear and well-arranged manner instructions to the nurse whose job it is to carry out her part in the occupational work of the hospital. The author shows " that Occupational Therapy benefits all types of patients, excepting, of course, those who are gravely ill or in need of absolute rest, and that extensive facilities are required for providing it," and she reviews the special requirements of each type of patient, from the habit training of the most deteriorated patient to the elaborate handwork of the intelligent and cultured men and women whose minds are temporarily disordered. For those who wish to realize the complete change of atmosphere brought about in the mental hospital by the occupation officer's work, this booklet is strongly recommended.

**Emergency Surgery.** Third Edition. By HAMILTON BAILEY, F.R.C.S. Pp. xii., 852. Illustrated. Bristol: John Wright & Sons Ltd. 1938. Price 50s.—The young surgeon faced frequently with the treatment of urgent injury or illness is often glad to turn to an authoritative text-book for light on his problems. To this author's manual he will not turn in vain, for it includes all the standard procedures and also the latest advances in technique. Where necessary, the method advocated is supplemented by sound reasoning or by citing cases to exemplify the technique. Everything is told in lucid and succinct diction and the story is enriched by admirable illustrations. The publishers are to be congratulated on the clarity of the letterpress. There is no need to refer in detail to the different sections of the book, which range through the whole realm of surgery, including ear, nose, throat (by Mr. Watson-Williams) and eyes: for this production, unlike so many text-books, is not good in parts only, but very good throughout. The volume affords an invaluable aid and one which, within its handy compass, is unsurpassed in providing accurate information for every student, qualified or unqualified.

**Thoracic Surgery.** By F. SAUERBRUCH and L. O'SHAUGHNESSY, F.R.C.S. Pp. viii., 394. Illustrated. London: Edward Arnold & Co. 1937. Price 50s.—The surgery of the thorax has made rapid strides of late years. Since much of it has been carried out in special hospitals and sanatoria the medical student is apt to see far too little of chest surgery during the course of his training. As a consequence of this it often happens that he has very rudimentary notions of the help which the surgeon is often able to give to the physician in the treatment of chest complaints. The same statement holds good of many general practitioners and of not a few general surgeons and physicians. As the result of this it is to be feared that many patients suffering from chest complaints fail to receive all the benefits which modern surgery might provide for them. The names of Sauerbruch and of

O'Shaughnessy are in themselves a guarantee that this volume will contain an authoritative exposition of thoracic surgery as it is practised to-day. It is much to be hoped that it will be widely read not only by those who themselves contemplate practising this branch of surgery, but by medical men generally. In it they will find much that is provocative of thought. Illustrations, printing and indexing are good and there is an excellent bibliography at the end of the book.

**Actinomycosis.** By Z. COPE, M.S., F.R.C.S. Pp.xii., 48. Illustrated. London: Oxford University Press. 1938. Price 15s.—Actinomycosis is by no means a rare disease, but it is just sufficiently uncommon to catch out the unwary; so it behoves every medical man to acquaint himself with its common characteristics. Mr. Cope marshals a good deal of evidence to show that, although most cases of human actinomycosis are due to an anærobic fungus of the kind commonly met with in carious teeth, infection by an ærobic type may occur. This type has most frequently been identified in actinomycosis of skin, lungs, and in the rare pyæmic form of the disease. Attention is drawn to the difficulty in isolating actinomyces, especially where mixed infection is present. Possibly some variation in the strains of infecting organisms may account for the extreme differences in response to treatment. It is of interest to note that "big jaw" and "woody tongue," which were at one time considered to be identical in origin, are caused, the former by the ray fungus, the latter by the gram-negative actino-bacillus. It is for the second condition that treatment by iodide of potassium was introduced and for which it is a specific. A few cases of this bacillary infection have been recorded in human beings. Iodine in some form is still the great stand-by, whether given by the mouth, preferably in milk or cream; intravenously as colloidal iodine; hypodermically in the form of "tiodine"; or even intratracheally as lipiodol when treating actinomycosis of the lungs. Surgery, X-rays and radium also play a very useful part at times. Mr. Cope has succeeded in producing an interesting and very readable monograph. It is well illustrated and contains some excellent coloured plates. A useful bibliography is appended and the book is well indexed.

**Neuro-ophthalmology.** By R. Lindsay Rea, M.D., M.Ch., F.R.C.S. Pp. xxii., 568. Illustrated. London: William Heinemann Ltd. 1938. Price 42s.—Mr. Lindsay Rea deserves warm congratulations for his book. While perhaps not of direct appeal to all practitioners, it is of great interest to a much wider field than the ophthalmologist alone. Neurologists, general physicians, and the neuro-surgeon will find here, accurately and clearly described, all the ophthalmological facts relevant to their subject. Illustrations are numerous and excellent, many of them original; they have been well reproduced and the publishers are to be congratulated on the general excellence of the book. Mr. Lindsay Rea's happy Irish style, so well known to his fortunate colleagues, is well shown in this book, which is very readable, in spite of the often technical nature of the discussions. The book is designed to be used as one of reference as well as ready information, and there is an extensive bibliography. The author has not felt tied too tiresomely to the precise nature of his subject, and has given much accessory information, mainly neurological or pathological. This saves the enquirer much labour in referring from one book to another. The whole field has been generously covered, even to the inclusion of a section on neuro-ophthalmological changes in head injury cases. It is difficult to select any section for especial praise, criticism or comment, there being no doubt that the whole book is authoritative and excellent.

**The Patient and the Weather.** By WILLIAM F. PETERSEN, M.D., with the assistance of MARGARET S. MILLIKEN, S.M. Vol. iv., pt. 3. Organic Disease. Surgical problems. Pp. xxxvii., 651. Ann Arbor, Michigan: Edwards Brothers, Inc. 1938. Price $10.—We have received for review this one volume of a work which is to be completed in five volumes. The object of this encyclopædic monograph is the relation of disease to meteorological environment. The Hippocratic dictum that during violent changes of the weather one should neither purge nor apply cautery or knife to the bowels appears to be the text on which the present volume is based. There are many instances quoted of acute abdominal catastrophes coinciding with sharp rises and falls of the barometer. The author's object is to emphasize " the fact that the human must adjust to every environmental change of the atmosphere with far-reaching physiological changes." Although the book is heavy reading there is a great deal of interesting matter which cannot be lightly dismissed. The format of the book is novel. For the most part the pages are photo-lithographed from perfect typescript. Quotations from previously published articles are also photo-lithographed, so that the amount of new type-setting needed is minimized. The publishers explain that this method represents an attempt to make the publication of scholarly and technical books in small editions pay for themselves by economies in printing and distribution. The venture is amazingly successful even if at first the sudden changes in type appear somewhat violent and trying. A thicker face of type in the typewritten sections would be more readable.

**Disorders of the Blood.** Second Edition. By L. E. H. WHITBY, C.V.O., M.C., M.A., M.D., F.R.C.P., D.Ph., and C. J. C. BRITTON, M.D., D.Ph. Pp. xii., 582. Illustrated. London: J. & A. Churchill Ltd. 1937. Price £1 1s.—The authors of this work have covered a very large and difficult field in a particularly successful manner. The title chosen aims at emphasizing the rarity of primary disease of the hæmopoietic system, and the fact that changes in the peripheral blood are usually a symptom of dysfunction in some other system of the body. A large part of the text is devoted to the anæmias, which are treated from the point of view of cell-size and hæmoglobin concentration. Clear accounts of normal and abnormal erythropoiesis are given, and these, together with clinical data and methods of treatment, render the book of interest both from the medical and scientific standpoint. The story of the advance of knowledge regarding pernicious anæmia is well presented, and points of differentiation from anæmias due to hæmolysis and other causes are indicated clearly. Thrombocytopenia and the formation of thrombocytes are discussed, and the various leukæmic and leucopenic conditions are described, as are also the blood changes due to infection and infectious diseases, and the large part played in blood diseases by disfunction of the spleen. In all these complicated and little-understood fields the available information is presented with clarity and the limitations of theories and methods of treatment are assessed. The bibliography is well chosen and is remarkably up to date, while the coloured plates depicting normal and abnormal cells and bone-marrow add greatly to the value of the book. Most readers will be grateful for the summaries given to the chapters, and the appendix containing technical details of investigations should be found invaluable.

